# Mindfulness-based interventions and cognitive function among breast cancer survivors: a systematic review

**DOI:** 10.1186/s12885-018-5065-3

**Published:** 2018-11-26

**Authors:** Gabriella Cifu, Melinda C. Power, Sarah Shomstein, Hannah Arem

**Affiliations:** 1 Department of Epidemiology and Biostatistics, George Washington University, Milken Institute School of Public Health, Washington DC, USA; 2 Department of Psychology, George Washington University, Columbian College of Arts and Sciences, Washington DC, USA; 3 GW Cancer Center, 950 New Hampshire Avenue NW, Office 514, Washington DC, 20052 USA

**Keywords:** Mindfulness, Cognition, Cognitive decline, Mindfulness based stress reduction, Breast cancer, Survivorship

## Abstract

**Background:**

Breast cancer survivors have an elevated risk of cognitive impairment compared to age-matched women without cancer. Causes of this impairment are complex, including both treatment and psychological factors. Mindfulness-based interventions, which have been shown to improve cognitive function in the general population, may be one approach to mitigate cognitive impairment in this survivor population. Our objective was to conduct a systematic literature review of studies on the effect of mindfulness-based interventions on cognition among breast cancer survivors.

**Methods:**

We conducted searches of three electronic databases (Scopus, PubMed and Cochrane Database of Systematic Reviews) in September 2017 for studies pertaining mindfulness and cognitive function among breast cancer survivors. Abstracts were manually searched by two reviewers and additional articles were identified through reference lists.

**Results:**

A total of 226 articles were identified through our systematic search and six met inclusion criteria for this review. The reviewed studies lacked consistency in terms of the cognition domains studied (e.g. executive function, recent memory, etc) and in the measures used to assess cognition. Of the included studies, two found no association between mindfulness interventions and cognitive function, two found improvement that was not sustained at the follow-up, and another two found sustained improvement at 2- or 6-months.

**Conclusions:**

Mindfulness-based interventions have shown some evidence for improving cognition among breast cancer survivors, but further research using validated and comprehensive cognitive assessments is needed. More research is also needed related to the timing, duration and content of mindfulness interventions.

**Electronic supplementary material:**

The online version of this article (10.1186/s12885-018-5065-3) contains supplementary material, which is available to authorized users.

## Background

Breast cancer is the most common cancer among women and will account for an estimated 255,180 incident cancer cases in the United States in 2017 [1]. Survival rates are high for breast cancer patients, such that the average 5-year survival rate for all stages is 90% [1]. Among the late and long-term side effects of cancer diagnosis and treatment, cognitive change has been recognized as a concern in women with breast cancer, with lower performance than age-matched peers [2, 3]. Recent guidelines suggest a focus on cognitive domains that may be particularly affected, which include short- and long-term memory, cognitive processing speed (as opposed to perceptual processing speed), attention and concentration as a component of executive function, language, and cognitive control [4–8]. Causes of cognitive decline are complex and may include stress following cancer diagnosis, anxiety, depression, fear of recurrence or the effects of treatment, among other factors [9–12]. Additionally, studies suggest that chemotherapy and radiotherapy may increase risk of cognitive impairment in breast cancer patients, with “chemobrain”, or fogginess in thinking or memory, widely discussed as a side effect [5, 11, 13, 14].

The body of literature on cognitive changes among cancer survivors is growing, with emerging interventions to address cognitive decline [4, 15]. One type of intervention to mitigate cognitive impairment symptoms is mindfulness-based interventions. Here, we define mindfulness as practices that increase awareness of one’s body, mental state, and surroundings in the present moment, as well as the ability to control attention [16]. Mindfulness interventions incorporate “intentional and non-judgmental awareness of the present moment” [15] and may be a low-cost, non-pharmacological approach to multi-symptom treatment that may be practiced individually and outside of a clinical setting [15, 17–19].

**Fig. 1:**
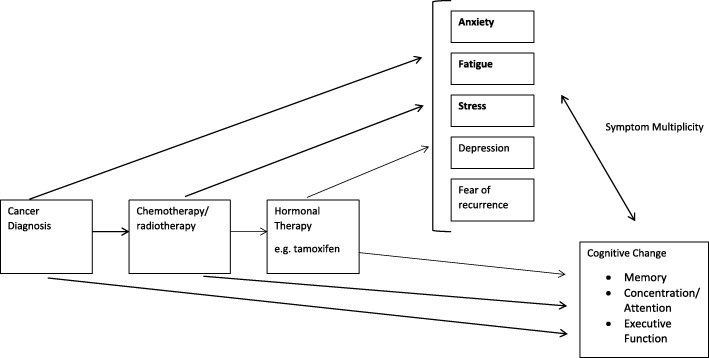
Conceptual Model for Understanding the Association Between Breast Cancer and Cognitive Impairment. Pathways though with mindfulness interventions could affect cognitive change

There has been preliminary evidence suggesting that mindfulness can improve cognition specifically in the domains of focused attention, working memory capacity, and other executive functions, although Chiesa et al. were not looking specifically at cancer survivors [19]. A 2014 systematic review found that mindfulness-based approaches to reducing cancer-related cognitive impairment may be effective, but could not be determined based on available research [18]. However, there has been more recent research showing that mindfulness-based stress reduction can significantly improve cancer-related cognitive impairment in breast and colon cancer survivors [17]. Given the complexity of causes of cognitive impairment among breast cancer survivors, we use Fig. 1 to illustrate our hypothesized potential relationships whereby mindfulness may affect the association between breast cancer diagnosis, treatment, and cognitive impairment.

The purpose of this review is thus to summarize the existing body of literature on mindfulness-based interventions and cognition in breast cancer survivors. Specifically, in breast cancer survivors, there is evidence to suggest that mindfulness-based interventions, compared to standard group therapies, can effectively reduce cognitive impairment after cancer diagnosis and treatment. Given the heterogeneity in cognitive assessment in the published studies, we gave particular attention to methods of cognitive assessment.

## Methods

In order to better understand the existing body of literature on the relationship between mindfulness interventions and cognitive change in breast cancer survivors and to identify gaps in the research, we conducted a systematic literature search using three electronic databases in September 2017: Scopus, PubMed and Cochrane Database of Systematic Reviews. Electronic search terms used in PubMed were:((((((“mindfulness”[MeSH Terms]) OR “mindfulness based stress reduction”[Title/Abstract]) OR “mbsr”[Title/Abstract]) OR “mindfulness meditation”[Title/Abstract]))) AND ((((((“breast tumor”[Title/Abstract]) OR ((“breast neoplasms”[MeSH Terms]) OR “breast cancer”[Title/Abstract]))))) OR “breast cancer survivor”[Title/Abstract]).

This review follows guidelines by the Preferred Reporting Items for Systematic Reviews and Meta-Analyses (PRISMA) Statement [20]. There were no date restrictions in our search. Duplicate articles were removed and sub-cohort studies were excluded if their parent study was included. Non-English articles and grey literature were excluded. Articles were then manually searched and eligible for inclusion if they (i) included breast cancer survivors, here defined as an individual living post-breast cancer diagnosis [21], as the primary study population (reflecting greater than 50% of study participants), (ii) reported on the impact of a mindfulness intervention, and (iii) had a primary outcome related to cognition or cognitive impairment. Reference lists of included and related studies were manually searched to ensure that the electronic databases had not missed any relevant studies. Due to the fact that there are relatively few studies on this subject, we did not limit inclusion by study design. Both authors (GC and HA) agreed upon included articles. GC extracted data into Table 1 that included study population, study design, specific mindfulness intervention used, control group, time since diagnosis or treatment, follow-up timing, outcome measurement of cognition, association measured, and study conclusions. This review was intended as a narrative summary of the literature given the small number of included studies and variation in cognitive assessment, and thus we did not plan or estimate a statistical summary measure of study results.

**Table 1:** Characteristics of Included Studies- Study Design and Participant Characteristics

Study	Participants	Study Design	Intervention	Controls	Time Since Diagnosis or Treatment
Reich et al. (2017) [25]	322 Stage 0-III post-treatment female breast cancer survivors	RCT	MBSR(BC)	UC	Treatment: 0.66 ± 0.51 years
Rahmani et al. (2015) [24]	24 stage I-III female breast cancer patients	RCT	MBSR and group conscious yoga	UC	Not stated
Rahmani et al. (2014) [23]	36 stage I-III female breast cancer patients	RCT	1. MBSR 2. Metacognition treatment (MCT)	UC	Not stated
Lerman et al. (2011)	68 female post-treatment cancer patients (approx. ~ 70% breast cancer)	RCT	MBSR	Waitlist control	Diagnosis: 3.9 ± 5.1 years
Johns et al. (2015) [17]	71 stage 0-III post-treatment breast and colorectal cancer survivors (BC *n* = 60)	RCT	MBSR	Fatigue education and support (ES)	Treatment: 2.4 years
Dobos et al. (2015) [26]	117 cancer patients and survivors participant in day-center care (91% female, 65% breast cancer)	Prospective single-arm cohort study	MBSR, naturopathic self-regulation and self-care, Mediterranean diet	n/a	Diagnosis: 2.3 ± 3.88 years

*MBSR* Mind-Body Stress reduction, *MBSR(BC)* Mind-Body Stress Reduction for Breast Cancer, *RCT* Randomized controlled trial, *UC* Usual Care, *SD* Standard deviation

Two authors (GC and HA) independently evaluated the studies for risk of bias using the NIH Quality Assessment of Controlled Intervention Studies or the Quality Assessment Tool for Before-After Studies With No Control Group as appropriate. The risk of bias assessment included the following domains: randomization method, allocation concealment, blinding of participants, providers and outcome assessments, homogeneity of study groups, follow-up, reliability/ validity of outcome measures, power, and use of an intent-to-treat analysis. Studies were rated as “good”, “fair” or “poor”, based on responses to each criterion. Differing assessments were discussed until consensus was reached.

The authors declare no conflict of interest and since publicly available, de-identified data was used, did not seek IRB approval for this systematic review.

## Results

### Study characteristics

**Fig. 2:**
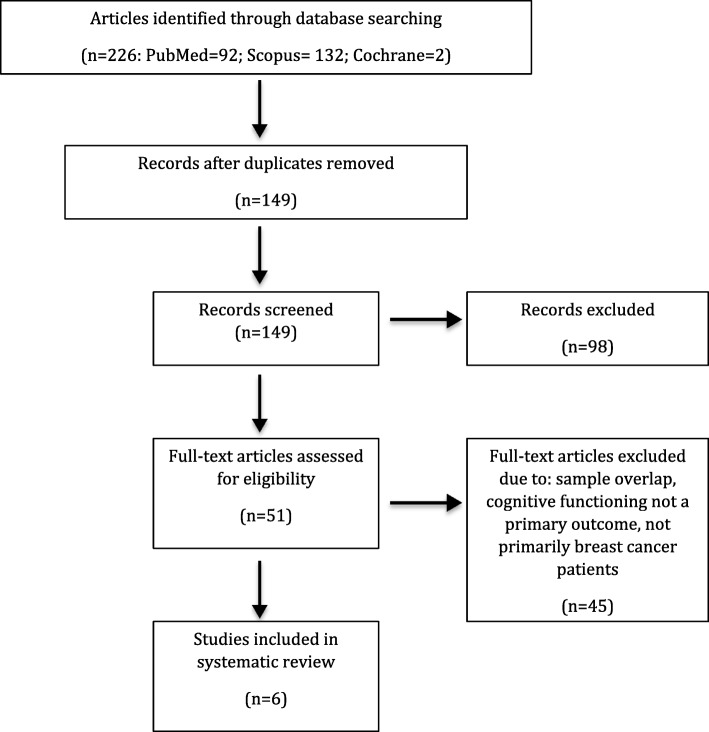
PRISMA flow diagram of literature search process

We included studies that used mindfulness as the primary intervention and assessed impact on cognitive change in breast cancer survivors. A total of six studies were included in this review (Fig. 2). Of the included studies, five used a randomized controlled design [17, 22–25]. The sixth study was a prospective cohort study comparing pre- and post- scores in a group of women participating in a mindfulness-based stress reduction intervention (MBSR) [26]. Studies included were conducted in the United States, Germany and Iran. Control groups included usual care (UC) [23–25], waitlist controls [22], and fatigue education and support (ES) [17]. The sample size ranged from 24 to 322 breast cancer survivors. The mean age of mindfulness group participants was 53.1 years. Mean time since diagnosis was reported for two [22, 26] of the studies (2.3 ± 3.88 and 3.9 ± 5.1 years), while mean time since treatment was reported in two [17, 25] other studies (0.66 ± 0.51 and 2.4 years). The last two studies reported neither elapsed time measure [23, 24]. Five of the studies only included survivors of non-metastatic breast cancer [17, 23–26], while the sixth included all post-treatment breast cancer survivors [22]. Follow-up time occurred at post-intervention (6, 8 or 11 weeks) in all of the studies, as well as 2, 3 or 6-months post-intervention in five of the studies [17, 23–26]. Study characteristics are summarized in Tables 1 and 2.

**Table 2:** Characteristics of Included Studies- Outcomes Measures

Study	Follow-up	Outcome Measurement (Cognition)	Results	Conclusions
Reich et al. (2017) [25]	Post-intervention (6 weeks) and 12 weeks follow-up	ECog; CAMS-R	ECog Mean (SD) in MBSR(BC) vs. UC; Baseline: 1.75 (0.88) vs. 1.65 (0.74), Week 6: 1.47 (0.71 vs. 1.51 (0.74), Week 12: 1.41 (0.68) vs. 1.50 (0.72)	No significant differences between groups in terms of the cognitive cluster. Significant differences in psychological and fatigue clusters
Rahmani et al. (2015) [24]	Post-intervention (8-weeks) and 2 months post-intervention	EORTC QLQ-C30	Cognitive Function Mean (SD) in MBSR vs. UC; Pre-test: 62.50 (10.4) vs. 59.72 (8.6) vs. Post-test: 75.00 (11.2)** vs. 59.72 (11.1), Follow-up 72.22 (13.0) vs. 61.11 (16.4)	Significantly improved cognition at the post-test follow-up in the MBSR group; improvements not sustained at the 2-month follow-up
Rahmani et al. (2014) [23]	Post-intervention (8 weeks) and 2-months post-intervention	EORTC QLQ-C30	Pre-test, post-test, and follow-up mean differences of cognitive function between MBSR and UC (2.78, 15..28**, 13.89*), between MCT and UC (− 13.89**, 1.39, 0.00) and between MBSR and MCT (16.67***, 13.89*, 13.89*)	Cognitive function increased from the pre-test to the post-test in the MBSR program; results remained relatively stable at follow-up
Lerman et al. (2011)	Post-intervention (8 weeks)	SOSI, EORTC QLQ-C30	Mean difference in cognitive disorder pre-test vs. post-test: 1.04 (*p* = 0.052)	The cognitive disorder score difference showed improvement in cognition and approached significance; suggests MBSR improved symptoms and QOL
Johns et al. (2015) [17]	Post-intervention (8 weeks) and at 6-month follow-up	AFI, Stroop Test	AFI total and Effective Action at T1, T2, T3 for MBSR vs. UC; (48.00, 64.64***, 64.83***) vs. (45.83, 52.28*, 55.21**) and (44.88, 63.16***, 62.74***) vs. (43.05, 50.09, 52.53*)	Both groups improved over time, but MBSR participants showed significantly greater and sustained improvements on AFI total score and most subscales.
Dobos et al. (2015) [26]	Post-intervention (11 weeks) and 3-months post-intervention	EORTC QLQ-C30	Pre-, post-, follow-up cognitive function score means (SD); 62.68 (29.2)***, 72.25 (27.0)***, 70.10 (27.7)***	Improvements in cognitive function, sustained at 6-month follow-up. Mindfulness-based interventions considered effective to improve physical and mental health.

*ECog* Everyday Cognition Scale, *CAMS-R* Cognitive and Affective Mindfulness Scale- Revised, *EORTC QLQ-C30* European Organization for Research and Treatment of Cancer Quality-of-Life Questionnaire, *SOSI* Symptoms of Stress Inventory, *AFI* Attentional Function Index

*p < 0.05

**p < 0.01

***p < 0.001

Generally, rates of retention were high in the included studies. Retention in MBSR intervention groups ranged from 83.8–94.3%, with comparable rates of retention in the control groups [17, 22, 25, 26]. Two studies did not include information on loss-to- follow-up [23, 24]. Still, future studies should continue to consider methods to recruit and retain participants.

### Describing the mindfulness interventions

In five of the included intervention studies, participants were randomized to receive mindfulness therapy or a control therapy, which ranged from wait-list control to cognitive, psychosocial, or fatigue education. Four of the programs utilized the MBSR program developed by Kabat-Zinn [17, 22–24]. MBSR was developed for chronic pain and anxiety, and consists of 8 weekly 2-h long classes [27]. Each class contains elements of controlling and self-regulating attention, in order to help control and reduce stress and associated symptoms. Participants are given education materials on mind-body practices and mediations, are instructed to practice meditation during weekly sessions and at home, and receive practice tools to reduce barriers to mindfulness. Attention on the breath, focused body-scans, Hatha yoga, and walking meditation are also emphasized as mind-body practices. Additionally, in traditional MBSR, a 7.5-h meditation retreat takes place during the sixth week of the intervention. Lengacher et al. adapted the widely-used MBSR program for breast cancer survivors (MBSR-BC) [28] which was used by Reich et al. study [25]. Similar in structure to MBSR, MBSR(BC) involves 2-h sessions for 6 to 8 weeks that focus on the same elements of attention and attention control. The modified program also encourages 15–45 min of formal meditation 6 days a week, as well as 15–45 min of informal meditation while doing usual activities (e.g. walking, driving, or eating). Participants are given audio recordings of daily, guided meditations and are asked to keep a daily meditation diary. MBSR(BC) does not always include a meditation retreat [29]. Doctoral level clinical psychologists or physicians with MBSR experience instruct both versions of this mind-body intervention. There is no specified group size, but the majority of groups range from 10 to 15 participants.

The other study included in our review combined elements of MBSR, naturopathic and self-care methods and a Mediterranean diet, during a weekly semi-residential 6-h session once weekly over 11 weeks [26]. This program was designed to include an entire MBSR course, including the mindfulness retreat, in addition to the other elements, to create a longer and more intensive intervention. MSc- or PhD-level health professionals, who are trained in MBSR and psychosocial counseling, delivered the program.

### Tools for measuring cognition among breast cancer survivors

A major challenge in comparing and summarizing studies of cognitive function in cancer survivors stems from variation in cognitive measurement. There is no single validated scale for self-reported cognition assessment in breast cancer patients, and 2011 research recommendations suggest objective tests of cognition or subjectively assessing mood and fatigue may more adequately measure cognitive impairment [6]. The tools used in the studies included in this review are summarized in Table 3 including example questions. Self-report measures included the European Organization for Research and Treatment of Cancer Quality of Life Questionnaire - C30 (EORTC QLQ-C30) survey [22–24, 26], Cognitive and Affective Mindfulness Scale (CAMS) [25], Everyday Cognition Scale (ECog) [25], Attentional Function Index (AFI) [17], and the Calgary Symptoms of Stress Index (C-SOSI) [22].The EORTC QLQ-C30 is designed for cancer patients in clinical trials and includes a cognitive functioning sub-scale with two questions assessing difficulty with concentration and memory [30]. The CAMS is used to conceptualize and report on mindfulness, with questions specific to concentration and attention regulation [31]. The informant-rated ECog scale has been validated against the Blessed Dementia Rating Scale (BDSR), Clinical Dementia Rating Scale (CDR), Mini-Mental State Exam (MMSE), and clinical diagnosis records, and includes 6 sub-factors (everyday memory, language, visuospatial abilities, planning, organization, and divided attention) and one global factor, to create an assessment of everyday cognitive function [32]. The AFI assesses self-perceived selective attention and working memory in completing daily activities [33]. Last, the C-SOSI was designed to measure multiple domains of stress in a concise manner, and includes a sub-scale evaluating cognitive disorganization [34]. The study by Reich et al. specifically sought to evaluate the relationship between symptom assessment tools and four symptom clusters (pain, psychological, fatigue and cognition), and reported a beta of 0.94 between the ECOG (memory scale) and the cognitive cluster, but a beta value of only 0.33 between the CAMS (total score) and the cognitive cluster, suggesting the importance of tool selection in evaluating cognition [25]. One study [17] also used a widely used neuropsychological test, the Stroop-Word Test, which was designed to evaluate inhibition of cognitive interference, as well as attention, processing speed, cognitive flexibility and working memory [35–38].

**Table 3:** Summary of Cognition Assessment Measures

Scale	Example Questions	Number of questions used to assess cognition	Domains of cognition tested	Validated? (y/n)	Measure validated against	*P*-value for validation assessemnt
Everyday Cognition (ECog) [34]	1. Remembering a few shopping items without a list. 2. Remembering things that happened recently (such as recent outings, events in the news). 3. Recalling conversations a few days later. 4. Remembering where she/he has placed objects. 5. Repeating stories and/or questions. 6. Remembering the current date or day of the week 7. Remembering he/she has already told someone something. 8. Remembering appointments, meetings, or engagements. 9. Verbally giving instructions to others. 10. Following a story in a book. (full list of questions available: https://www.ncbi.nlm.nih.gov/pmc/articles/PMC2877034/pdf/nihms-200864.pdf)	39 questions	Global cognition, Everyday memory, everyday language, everyday visuospatial, everyday planning, everyday organization, everyday divided attention	Y	Blessed Dementia Rating Scale (BDSR), Clinical Dementia Rating Scale (CDR), Mini-Mental State Exam (MMSE), Clinical Diagnosis	Global cognition *p* < 0.001 (accurately discriminating between clinical groups)
Cognitive and Affective Mindfulness Scale-Revised (CAMS-R) [33]	1. It is easy for me to concentrate on what I am doing. 2. I am preoccupied by the future. moment in considerable detail. 3. I am easily distracted. 4. I am preoccupied by the past. 5. I am able to focus on the present moment. 6. I am able to pay close attention to one thing for a long period of time.	6 (out of 12 questions)	Attention, present focus	Y	Cognitive Flexibility Scale (CFS), The Measure of Mental Anticipatory Processes (MMAP);	< 0.001
European Organization for Research and Treatment of Cancer Quality-of-Life Questionnaire (EORC QLQ-C30) [32]	1. Have you had difficulty in concentrating on things, like reading a newspaper or watching television? 2. Have you had difficulty remembering things?	2 (out of 30 total questions)	Cognitive function subscale	N	Psychosocial Adjustment to Illness Scale (PAIS), Profile of Mood States (POMS), Mental Adjustment to Cancer Scale (MAC), Impact of Event Scale (IES)	Non-significant
Calgary Symptoms of Stress Inventory (C- SOSI) [36]	1. You must do things very slowly to do them without mistakes 2. You get directions and orders wrong 3. Your thinking gets completely mixed-up when you have to do things quickly 4. You have difficulty in concentrating 5. You become suddenly frightened for no good reason 6. You become so afraid you can’t move	6 (out of 56 total questions)	Cognitive disorganization	N	EORTC QLQ C^−30^, POMS, Pittsburg Sleep Quality Index (PSQI)	*P* < .01 for cognitive subscales on EORTC QLQ C-30 and POMS, and global PSQI score
Attentional Function Index (AFI) [35]	1. Getting started on activities 2. Following through on plans 3. Doing things that take time and effort 4. Making mind up about things 5. Keeping mind on what you are doing 6. Remembering to do the things you started out to do 7. Keeping mind on what others are saying 8. Keeping self from saying or doing things 9. Being patient with others 10. How hard you find it to concentrate on details 11. How often you make mistakes 12. Forgetting important things 13. Getting easily annoyed/irritated	13 questions	Effective action, attentional lapses, interpersonal effectiveness	Y	Symptom Distress Scale (SDS), Cognitive Failures Questionnaire (CFQ), POMS	*P* < 0.01 (AFI and CFQ; improved perceived attention scores associated with decreased reported cognitive failures); *p* < 0.01 for AFI and POMS confusion subscale (increased AFI scores associated with decreased feelings of confusion)
Stroop Color and Word Test [37–40]	Neuropsychological assessment: word reading, color naming, named color-word	–	Inhibition of cognitive interference	Y	fMRI, clinical diagnosis	–

*ECog* Everyday Cognition Scale, *CAMS-R* Cognitive and Affective Mindfulness Scale- Revised, *EORTC QLQ-C30* European Organization for Research and Treatment of Cancer Quality-of-Life Questionnaire, *SOSI* Symptoms of Stress Inventory, *AFI* Attentional Function Index, *BDSR* Blessed Dementia Rating Scale, *CDR* Clinical Dementia Rating Scale, *MMSE* Mini-Mental State Exam, *CFS* Cognitive Flexibility Scale, *MMAP* The Measure of Mental Anticipatory Processes, *PAIS* Psychosocial Adjustment to Illness Scale, *POMS* Profile of Mood States, *MAC* Mental Adjustment to Cancer Scale, *IES* Impact of Event Scale, *PSQI* Pittsburg Sleep Quality Index, *SDS* Symptom Distress Scale, *CFQ* Cognitive Failures Questionnaire

### Outcomes

In the included studies, mindfulness interventions had heterogeneous impacts on cognition (Table 1). One study showed no significant differences comparing post-intervention cognitive cluster scores between the mindfulness and control groups [25], while another study showed no significant differences in improvements to cognitive disorder between the mindfulness intervention and waitlist controls groups (*p* = 0.052) [22]. Two studies showed significant cognitive improvements in mindfulness intervention groups in a pre-and-post test assessment compared to usual care. Improvements were sustained to a lesser extent at the two (η = 0.20) [24] or three (R^2^ = 0.234) [26] month follow-up. Two other studies showed significantly improved cognition in the mindfulness group compared to control groups, with results sustained at both two- (ηp^2^ = 0.35) [23] and six- month (Cohen’s *d* = 0.55) [17] follow-ups. No studies reported significant differences by group at baseline, with the exception of greater use of anxiolytic medications in the MBSR(BC) group in the study by Reich et al [25].

Johns et al. also noted that cognitive function improved over time in both the MBSR and fatigue education/support groups, but that the MBSR participants experienced greater improvements and that the improvements were sustained at the six-month follow-up [17]. In the two studies showing sustained improvement, cognition was assessed using the EORTC QLQ-C30 [23] and the AFI [17]. Cognitive impairment in studies utilizing the EORTC QLQ-C30 specifically assessed attention and memory, based on two questions within a larger scale. Meanwhile, significant results on the AFI suggested improvement in both effective action and attentional lapse, as well as on overall attentional function.

Furthermore, using the Stroop-Word test, Johns et al. found that women in the MBSR group made fewer errors compared to the fatigue education group both at the eight-week post-intervention assessment at the six-month follow-up, suggesting sustained improvement in accuracy over time [17]. Stroop reaction time did not differ between groups. Furthermore, in the study conducted by Lerman et al. no significant improvement was noted for the cognitive disorganization (attention, planning, and organization) subscale of the SOSI [22].

Of note, the study by Reich et al. examined symptom clusters and found that although there were no significant improvements in the cognition symptom cluster, which consisted of everyday memory and an overall cognition and mindfulness score, MBSR did result in significant improvements in the fatigue and psychological symptom clusters, which may impact cognitive impairment in breast cancer patients [25]. Reich et al. commented on the potential use of MBSR to address multiple co-occurring symptoms, with the added benefit of individuals being able to practice techniques on their own [25]. The authors emphasized the importance of understanding related symptoms and managing multiple symptoms to improve overall well-being.

### Risk of Bias assessment

Three of the included studies [17, 25, 26] received a rating of “good”, one study [22] received a rating of “fair”, and the other two studies [23, 24] received a rating of “poor.”

The study by Lerman et al., rated as “fair,” did not have any major shortcomings, but there was insufficient information to answer all of the questions on the risk of bias assessment. Of note, the “poor” studies both reported associations between mindfulness and cognitive function improvement.’ The results of the risk of bias assessments are included in Additional file 1: Table S1A and B.

## Discussion

In our results, four of the included studies showed beneficial impacts of mindfulness interventions on cognitive function, while two showed no association. Thus, while this systematic review suggests some evidence for mindfulness interventions on cognitive function among breast cancer survivors, the lack of consistent direct measurement tools in this review precluded a meta-analysis of published results.

Included studies were also limited by their relatively small sample size (four had an *n* < 100) and by wait list control designs, which may not capture the differences in attention between groups. The effect size from 0 [22, 25] to *d =* 0.55 [17], but, as mentioned, outcome assessment tools varied by study. Additionally, while half of the included studies were rated “good,” the risk of bias assessment suggested caution in interpreting results of the included studies as the remaining studies had several design shortcomings or missing details, which limit the weight of the observed associations.

Many studies identified in our search but not included in the review did not assess cognition as a primary outcome, but rather focused on quality of life or other psychosocial variables as a primary outcome. Numerous studies on mindfulness in breast cancer survivors measure indirect psychological factors such as fear of recurrence/worry, perceived stress, anxiety, depression and fatigue to understand the cognitive challenges following cancer diagnosis and treatment. However, the relationship between these variables and cognition is not fully understood. Including all these proxy outcomes was beyond the scope of this review, but including measurements of factors outlined in Fig. 1 that may contribute to cognitive decline is important to include in future studies to better describe this association.

Cognitive impairment in cancer survivors has been shown specifically in the domains of memory, attention/concentration, information processing speed and executive function [4, 5, 39–43]. Thus, neuropsychological assessments of cancer survivors generally focus on evaluating these domains both at the time of cancer diagnosis and after treatment, namely chemotherapy [44]. Cognitive impairment among breast cancer survivors may be persistent, as shown by a study that found that ten years post-treatment, women who had undergone chemotherapy had significant cognitive impairment in planning performance (an executive function task) and in a test of paired associates (attentional processing) compared to breast cancer survivors for whom chemotherapy was not prescribed [40]. In this study, functional magnetic resonance imaging (fMRI) suggested that deficits in executive functioning were related to decreased selective activation of the dorsolateral prefrontal cortex and parahippocampal gryus, while deficits in attentional processing were associated with a decrease in activation of the lateral posterior parietal cortex [40]. Another study examining fMRI of breast cancer survivors treated with chemotherapy suggested that chemotherapy may specifically affect executive function by reducing activity of the left caudal lateral prefrontal region, compared to breast cancer patients who had not received chemotherapy and healthy women [45].

While there is a large body of evidence to support “chemobrain,” studies have also found cognitive changes at the time of cancer diagnosis and prior to initiation of chemotherapy. One such study found that out of 12 different cognitive tests, women with breast cancer who had not yet received chemotherapy scored worse on five tests than standardized norms, adjusting for anxiety and depression [11]. These five tests included the D2 test (concentration), Trail Making Test Part B (psychomotor function, divided attention and cognitive flexibility), and three Regensburg Word Fluency Test subtests: lexical search, semantic search and lexical search with change of category.

Another proposed contributor to early decline in cognitive function is the stress of a cancer diagnosis. While the mechanism by which stress affects cognitive function is not well understood, research has suggested that chronic stressors can reduce prefrontal cortex glutamatergic synaptic transmission, which can negatively affect cognitive processes that are dependent upon the prefrontal cortex [12]. Thus repeated acute stress may be associated with decreased working and recognition memory. An additional study has suggested that cognitive impairment may be due to neurotoxic or microvascular injuries, inflammation, regulatory issues with the hypothalamic-pituitary-adrenal axis that could impact brain hormone concentrations, or increases in the rate of cellular aging, as a result of cancer tumor or treatment [46].

Attention and arousal are also commonly evaluated in assessing cognitive function. The brain’s locus coeruleus-norepinephrine system is associated with arousal and attention, as well as cognition. The coeruleus-norepinephrine system controls both arousal and pupil dilation, the latter of which has been proposed as a method of evaluating cognition [47]. Other studies have suggested that Group II Metabotropic Glutamate Receptors, which are associated with control of synaptic transmission and neuronal excitability, may also help explain the relationship between arousal and cognition, and how improvements in attention through focused mind-body activities can also predict improved cognitive processes [48, 49]. Additionally, various aspects of attention rely more heavily on cognitive processes than others (e.g., top-down sensitivity versus bottom-up signaling), which is an important distinction to be made in research assessing cognition via attention or arousal [50].

Studies on mindfulness-based interventions that assess cognition as a primary outcome often do not distinguish between the various domains and focus on only a summary measure of cognitive function, or use measures of self-reported cognitive difficulties, which may indicate other issues of or in addition to cognitive impairment (e.g. depression, personality) [18, 51, 52], limiting our understanding of explanatory mechanisms. Still, Johns et al. found that women in the MBSR group showed significant improvement in both attention and executive function (e.g. processing speed) domains using the AFI and Stroop-Word Test, although the mechanisms, which may be different for each domain, could not be assessed in the study [17].

There is ongoing research to address some of these gaps. For example, Lengacher is testing the impact of MBSR(BC) on executive functioning using the Stroop Neuropsychological Screening Test, with secondary outcomes of visuospatial, verbal, logical memories, attention and concentration and verbal fluency (5R01CA199160–03) [53].

Future studies would benefit from more homogeneous outcomes assessments of cognition that are sensitive to the changes reported by cancer patients, particularly using those scales with proven validity and reliability. It is also important to understand what components of mindfulness interventions target various domains of cognition (attentional selection, working memory, active maintenance, cognitive processing speed, etc.) to inform intervention design overall, as well as intervention design for specific types of cognitive dysfunction. Specifically, we need more sensitive measures of cognition that are appropriate to breast cancer survivors, who tend to be non-demented and highly functional. Furthermore, we need these measures to be administered in a way that is adequately powered and allows for meta-analysis.

There are many potential benefits of mindfulness-based interventions in terms of feasibility and delivery options. In addition to being relatively low-cost, mindfulness-based interventions can be successfully utilized in more heterogeneous populations, specifically in terms of severity of diagnoses and demographics [54]. Recent literature has also suggested that mindfulness-based interventions may have increased benefit to more vulnerable health populations and may better meet the needs of more culturally diverse populations than current group therapies [54–56]. Additionally, while mindfulness-based interventions stress the important of frequent practice, literature has suggested that participants may still see benefit without extensive home practice, which may have further implications in terms of feasibility and may warrant additional study [54, 57].

Studies on mindfulness in healthy individuals suggest an impact on neurogenesis, synaptogenesis, or dendritic branching, in addition to preserving and preventing the apoptosis of neurons [16]. Neuroimaging studies have suggested that mindfulness training induces changes in regions including the medial cortex, insula, amygdala, basal ganglia, and lateral frontal regions, specifically decreasing signaling in the bilateral anterior insula, left ventral anterior cingulate cortex, right medial prefrontal cortex, and bilateral precuneus and increasing signaling in the right posterior cingulate cortex [58, 59]. There is also evidence to suggest that mindfulness can affect the three neural networks of attention (alerting, orienting and executive), self-referential thinking, and emotional regulation [59]. However, studies of multiple neural systems need to be conducted to fully understand the mechanism by which mindfulness interventions can result in changes in the brain and lessening cognitive decline or improving cognitive impairment, particularly in the cancer survivor population.

A future challenge in measuring mindfulness is how to capture achieved intervention “dose”. Participants may be asked to track time spent in mindfulness activities, but it is difficult to measure the achieved time spent in the activity, especially if it is spread throughout the day. Furthermore, there is little evidence outside the MBSR intervention about mindfulness and cognitive outcomes. More research is needed to determine whether specific practices or durations have the greatest impact on cognitive function. Larger randomized control trials that rigorously test the effects of mindfulness interventions on cognition and specific mechanisms of action are also needed, including studies using objective fMRI measures. Future studies should also focus on short and long- term effects of mindfulness on cognition, as improvements were not always sustained post-intervention.

## Conclusions

Mindfulness based interventions show some evidence for improving cognitive impairment among breast cancer survivors. Still, this review highlights the need for the consistent use of scales assessing multiple domains of cognition (e.g. the ECog and AFI scale), complimented by standard neuropsychological tests, as well as measurement and adjustment for potential mediating or confounding factors.

## Additional file

Additional file 1: **Table S1A.** Quality Assessment of Controlled Intervention Studies. **Table S1B.** Quality Assessment Tool for Before-After (Pre-Post) Studies With No Control Group. (DOCX 67 kb)

## Abbreviations

AFI: Attentional Function Index
BDRS: Blessed Dementia Rating Scale
CDR: Clinical Dementia Rating Scale
C-SOSI: Calgary Symptoms of Stress Index
ECog: Everyday cognition scale
EORTC QLQ-C30: European Organization for Research and Treatment of Cancer Quality of Life Questionnaire - C30
fMRI: Functional magnetic resonance imaging
MBSR: Mindfulness-based stress reduction
MMSE: Mini-Mental State Exam
PRISMA: Preferred Reporting Items for Systematic Reviews and Meta-Analyses
