# Supplementary material for: Mindfulness-based interventions and cognitive function among breast cancer survivors: a systematic review
Source: BMC Cancer. 2018 Nov 26;18:1163. doi: 10.1186/s12885-018-5065-3 (PMC6260900; doi:10.1186/s12885-018-5065-3)
Supplement: Supplementary file 1 — Table S1A. Quality Assessment of Controlled Intervention Studies. Table S1B. Quality Assessment Tool for Before-After (Pre-Post) Studies With No Control Group. (DOCX 67 kb) [file 12885_2018_5065_MOESM1_ESM.docx]

Supplemental Tables

Table S1A: Quality Assessment of Controlled Intervention Studies

|  | Reich et al.  (2017) | Rahmani et al. (2015) | Rahmani et al.  (2014) | Lerman et al.  (2011) | Johns et al.  (2015) |
| --- | --- | --- | --- | --- | --- |
| Described as RCT | + | - | - | + | + |
| Randomization | + | - | - | + | + |
| Treatment Allocation Concealed | + | - | - | U | + |
| Participants and Providers Blinded | - | - | - | - | + |
| Outcome assessors blinded | - | - | - | - | + |
| Homogeneity of baseline groups | + | + | + | + | + |
| Drop-out rate <20% | + | + | + | + | + |
| Differential drop-out rate <15% points | + | + | + | + | + |
| High adherence to protocols | + | + | + | U | + |
| Other interventions avoided | + | + | + | + | + |
| Outcomes assessed using valid/reliable measures | + | + | + | + | + |
| >80% power | - | - | - | + | - |
| Outcomes reported | + | + | + | + | + |
| Intent-to-treat analysis | + | - | - | U | + |
| Rating | **Good** | **Poor** | **Poor** | **Fair** | **Good** |

U=unclear; not addressed in study

Table S1B: Quality Assessment Tool for Before-After (Pre-Post) Studies With No Control Group

|  | Dobos et al.  (2015) |
| --- | --- |
| Study objective stated | + |
| Eligibility/ selection criteria | + |
| Study participation representative | + |
| All eligible participants enrolled | + |
| Sample size sufficient | + |
| Test/service/intervention clear and delivered consistently | + |
| Outcomes valid and reliable | + |
| Outcome assessors blinded to interventions | N/A |
| Loss to follow-up <20% | + |
| Appropriate statistical methods | + |
| Interrupted time-series design | + |
| Effects at group level | + |
| Rating | **Good** |
